# Chronic Hepatitis B with Spontaneous Severe Acute Exacerbation

**DOI:** 10.3390/ijms161226087

**Published:** 2015-11-26

**Authors:** Wei-Lun Tsai, Wei-Chi Sun, Jin-Shiung Cheng

**Affiliations:** 1 Division of Gastroenterology, Department of Internal Medicine, Kaohsiung Veterans General Hospital, Kaohsiung 813, Taiwan; wcsun@vghks.gov.tw (W.-C.S.); rcheng@ms2.hinet.net (J.-S.C.); 2 School of Medicine, National Yang Ming University, Taipei 100, Taiwan

**Keywords:** hepatitis B, acute exacerbation, antiviral treatment

## Abstract

Chronic hepatitis B virus (HBV) infection is a major global health problem with an estimated 400 million HBV carriers worldwide. In the natural history of chronic hepatitis B (CHB), spontaneous acute exacerbation (AE) is not uncommon, with a cumulative incidence of 10%–30% every year. While exacerbations can be mild, some patients may develop hepatic decompensation and even die. The underlying pathogenesis is possibly related to the activation of cytotoxic T lymphocyte-mediated immune response against HBV. An upsurge of serum HBV DNA usually precedes the rise of alanine aminotransferase (ALT) and bilirubin. Whether antiviral treatment can benefit CHB with severe AE remains controversial, but early nucleos(t)ide analogues treatment seemed to be associated with an improved outcome. There has been no randomized study that compared the effects of different nucleos(t)ide analogues (NA) in the setting of CHB with severe AE. However, potent NAs with good resistance profiles are recommended. In this review, we summarized current knowledge regarding the natural history, pathogenetic mechanisms, and therapeutic options of CHB with severe AE.

## 1. Introduction

Chronic hepatitis B virus (HBV) infection is a major global health problem with an estimate of 400 million HBV carriers worldwide [1]. In the natural history of chronic hepatitis B (CHB), spontaneous acute exacerbation (AE) is not uncommon, with a cumulative incidence of 10%–30% every year [2,3,4]. While exacerbations can be mild, some patients may develop hepatic decompensation and even die [5,6]. The Asian Pacific Association for the Study of the Liver (APASL) had a consensus recommendation on acute-on-chronic liver failure (ACLF), defined as an acute hepatic insult manifesting as jaundice and coagulopathy (international normalized ratio [INR] >1.5), complicated within four weeks by ascites and/or encephalopathy in a patient with previously diagnosed or undiagnosed chronic liver disease [7]. AE of chronic hepatitis B (CHB) represents a distinct disease characterized by the abrupt rise of HBV DNA followed by impairment of liver function. AE of CHB was defined as “an abrupt elevation of serum alanine aminotransferase (ALT) to >5 × ULN or a greater than 3-fold increase in ALT, whichever was higher” [2,8]. Initiating events for the AE in CHB may not be readily identifiable, and these flares are considered to be spontaneous in nature. However, in many instances, precipitating factors for reactivated hepatitis B can be readily identified and are not considered as spontaneous in nature. The precipitating factors include immunosuppressive/cytotoxic medications, anti-viral therapy (interferon, nucleos(t)ide analogues), HBV genotypic variations (precore, core promoter or polymerase mutants), superimposed with other hepatotropic viruses (hepatitis A virus, hepatitis C virus or hepatitis D virus), and interaction with HIV (reactivated hepatitis, effect of immune reconstitution therapy) [9,10,11].

CHB with spontaneous AE is a dynamic process of immune response between HBV, hepatocytes and immune cells of the host [8,10,12,13]. Persistent necroinflammatory changes in liver tissue during CHB with AE are caused by an inadequate immune response to HBV antigens that are expressed on the surface of hepatocytes where they can activate cell-mediated immune responses [14,15]. When the immunologic response to viral antigens are more robust, the likelihood of inflammatory changes, damage to hepatocytes, and progressive fibrosis are greater [5]. The role of antiviral agents in the treatment of CHB with severe AE is unclear. Early lamivudine treatment before the bilirubin level rose above 20 mg/dL seemed to be associated with an improved outcome [16]. Another recent study also found that lamivudine treatment can improve the outcome for patients with a model for end stage liver disease (MELD) score below 30 [17]. However, the choice of different antiviral agents remains controversial. In this review, we summarized the natural history, pathogenetic mechanism of CHB with spontaneous severe AE, and compared the effects of different antiviral agents.

## 2. Natural History

Typical chronic HBV infection acquired perinatally involves three phases: immune tolerant, immune clearance, and inactive residual [18,19]. Severe exacerbation of CHB usually occurs in the immune clearance phase but can also happen in some patients in the inactive phase, inducing immune-mediated liver injury that resembles the events in the immune clearance phase [19]. A prospective study from Taiwan found that during an average follow-up of 23.5 months, AE occurred in 197 HBeAg-positive patients and 56 anti-HBe-positive patients, with a calculated annual incidence of 28.6% and 10.3%, respectively [20]. Another prospective study from Hong Kong reported that the cumulative probabilities of developing exacerbations at the end of one and four years were 6.3% and 15%, respectively in patients with serum alanine aminotransferase (ALT) levels below 200 IU/L, 24% and 47%, respectively in patients with ALT levels above 200 IU/L, and up to 8% of patients may develop hepatic decompensation [21]. The clinical presentation of CHB with spontaneous AE varies from either asymptomatic or symptomatic to a feature similar to overt acute hepatitis. It may also complicate with hepatic decompensation and even lead to death [19,20,21]. CHB with AE may also occur in cirrhotic patients and is associated with a higher rate of hepatic decompensation and mortality compared with CHB patients without cirrhosis [22]. Predisposing factors of spontaneous severe AE include elevated ALT levels at presentation, male sex, and the presence of HBeAg [3]. Yuen *et al.* [23] suggested that the prognostic factors in CHB with spontaneous severe AE not receiving antiviral treatment included pre-existing cirrhosis, high Child-Pugh score, low albumin level, high bilirubin level, prolonged prothrombin time (PT), and low platelet count, while prognostic factors for subsequent monitoring were high peak bilirubin level, long peak PT, duration to reach peak PT, development of encephalopathy, and presence of ascites. The prognostic factors reported by Tsai *et al.* [24] included high AST level, low albumin level, high bilirubin level, prolonged PT, and low platelet count.

## 3. HBV Genotypes and Variants in Acute Exacerbation

HBV genotypes (Table 1) and mutations in the precore and core promoter region of the HBV genome (Table 2) have been suggested to be associated with AE of CHB. In two studies from Hong Kong, genotype B HBV was found to be the predominant HBV strain among patients with severe AE [25,26]. Imamura *et al.* [27] also reported that HBV genotype B occurred more frequently in patients with acute forms of liver disease than in patients with chronic liver disease, and more frequently in patients with fulminant hepatitis than in those with acute self-limited hepatitis. Ren *et al.* [28] found that patients with CHB infected with genotype B were more likely to develop HB-ACLF than those with genotype C. However, Tsai *et al.* [24] found no significant association of genotype B HBV in CHB with severe AE. Liu *et al.* [29] in a case control study found that the distribution of HBV genotypes in HBV carriers with fulminant and subfulminant hepatitis was similar to control patients. Yuen *et al.* [30] in a recent study also found that there were no differences in the cumulative risk and severity of acute exacerbation between patients with genotypes B and C. Omata *et al.* [31] reported that a point mutation at the A1896 was associated with the development of fulminant hepatitis. However, further clinical studies on precore and core promoter mutations did not have consistent results. While Yuen *et al.* [26] found no association between precore mutation of HBV and severe AE of HBV, Tsai *et al.* [24] reported that precore mutation of HBV had a protective effect on the occurrence of hepatic decompensation in CHB with AE. On the other hand, Yuen *et al.* [26] found that core promoter mutation of HBV is associated with severe AE of HBV, Yuan *et al.* [32] also found that core promoter mutations were independently associated with the occurrence of acute exacerbation after HBeAg seroclearance. Ren *et al.* [28] found that single mutations including T1753V (C/A/G), A1762T, G1764A, G1896A and G1899A were more frequently detected in patients with HB-ACLF than in patients with CHB and patients with precore mutation had increased risk of a fatal outcome. Kusumoto *et al.* [33] found that Mutations in the core promoter (A1762T/G1764A) and precore region (G1896A) were more frequent in patients with acute exacerbation of chronic hepatitis than acute hepatitis. But Tsai *et al.* [24] reported no significant association between core promoter mutation of HBV and severe AE of HBV, and Liu *et al.* [29] in a case control study also found that the distribution of precore and core promoter mutations in HBV carriers with fulminant and subfulminant hepatitis were similar to control patients. Ehata *et al.* [34] found that clustering changes in a segment of 16 amino acids (codon 84–99 from the start of the core gene) were present in all seven fulminant and severe exacerbation patients infected with adr subtype HBV and a different segment with clustering substitutions (codon 48–60) was also found in seven of eight fulminant and severe exacerbation patients infected with adw subtype HBV. Mutations in the core region may play an important role in the pathogenesis of HBV, and such mutations are related to severity of liver damage. Liu *et al.* [35] discovered that after exacerbation of CHB, about half of the patients were repopulated by a different viral variant and mean nucleotide change per genome was 0.2 at virologic peak but increased to 4.4 and 8.1 at and after biochemical peak respectively, which was likely an effect of immune selection. Liu *et al.* [36] also found that HBV viral strain in the serum reflects the intrahepatic strain of the AE and random reactivation of the original HBV pool, rather than a sequential evolution of one strain, causes the onset of repeated AE.

**Table 1:** Hepatitis B virus genotypes in acute exacerbation, NA: not analyzed.

Authors	Disease	Patient No.	Genotype (%)	*p*-Value
Chan *et al.*[25]	Severe icteric flare up	21	B (91%)	<0.001
	Asymptomatic carrier	31	B (39%)	
	Early cirrhosis	49	B (20%)	
	Decompensated cirrhosis	31	B (32%)	
Yuen *et al.* [26]	Hepatic decompneation	28	B (71%)	0.0001
	No hepatic decompensation	39	B (28%)	
Imamura *et al.* [27]	Acute hepatitis	45	B (31%)	<0.001
	Fulminent hepatitis	16	B (63%)	
	Chronic liver disease	531	B (12%)	
Ren *et al.* [28]	Acute on chronic live failure	75	B (31%)	0.009
	Chronic hepatitis B	328	B (17%)	
Tsai *et al.* [24]	Hepatic decompensation	20	B (70%)	0.346
	No hepatic decompensation	31	B (80%)	
Liu *et al.* [29]	Fulminent/subfulminent hepatitis B	18	B (78%)	
	Hepatitis B carrier	18	B (67%)	>0.05
Yuen *et al.* [30]	Acute exacerbation	NA	B (NA)	0.95
	No acute exacerbation	NA	B (NA)	
	Severe exacerbation	NA	B (NA)	0.12
	Mild exacerbation	NA	B (NA)	

**Table 2:** Hepatitis B virus variants in acute exacerbation.

Authors	Disease (Patient No.)	Variants (%)	*p*-Value
Ren *et al.* [28]	Acute on chronic live failure (75)	G1896A (45%)	0.038
		G1899A (16%)	
		A1762T (77%)	0.013
		G1764A (83%)	
		T1753V (28%)	<0.001
	Chronic hepatitis B (328)	G1896A (32%)	
		G1899A (6%)	<0.001
		A1762T (52%)	
		G1764A (54%)	0.012
		T1753V (16%)	
Tsai *et al.* [24]	Hepatic decompneation (20)	Precore mutant (60%)	0.046
		Core promoter mutant (55%)	
	No hepatic decompensation (31)	Precore mutant (65%)	0.747
		Core promoter mutant (42%)	
Omata *et al.* [31]	Fatal hepatitis B (9)	G1896A (100%)	<0.05
	Acute self-limited hepatitis B (10)	G1896A (0%)	
Yuen *et al.* [26]	Severe exacerbation (24)	Precore mutant (17%)	NS
		Core promoter mutant (25%)	
	Mild exacerbation (96)	Precore mutant (18%)	
		Core promoter mutant (60%)	0.004
	No exacerbation (96)	Precore mutant (14%)	
		Core promoter mutant (46%)	
Kusumoto *et al.* [33]	Acute exacerbation (36)	Precore mutant (58%)	<0.001
		Core promoter mutant (81%)	
	Acute hepatitis (36)	Precore mutant (6%)	<0.001
		Core promoter mutant (19%)	
Yuan *et al.* [32]	Acute exacerbation (56)	Precore mutant (38%)	0.12
		Core promoter mutant (86%)	
	Without acute exacerbation (145)	Precore mutant (51%)	0.003
		Core promoter mutant (64%)	
Liu *et al.* [29]	Fulminent/subfulminent hepatitis B (18)	Precore mutant (67%)	>0.05
		Core promoter mutant (17%)	
	Hepatitis B carrier (18)	Precore mutant (50%)	NS
		Core promoter mutant (17%)	

NS: non-significant.

## 4. Pathogenesis

**Figure 1:**
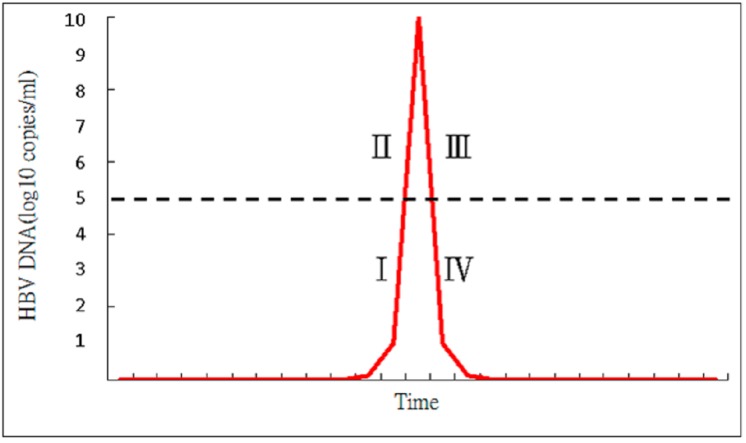
The clinical course of chronic hepatitis B (CHB) with acute exacerbation (AE) can be divided into four stages according to HBV DNA level. In the ascending limb, HBV DNA level <10^5^ copies/mL denotes Stage I while HBV DNA level ≥10^5^ copies/mL represents Stage II. In the descending limb, HBV DNA level ≥10^5^ copies/mL denotes Stage III while HBV DNA level <10^5^ copies/mL represents Stage IV.

**Figure 2:**
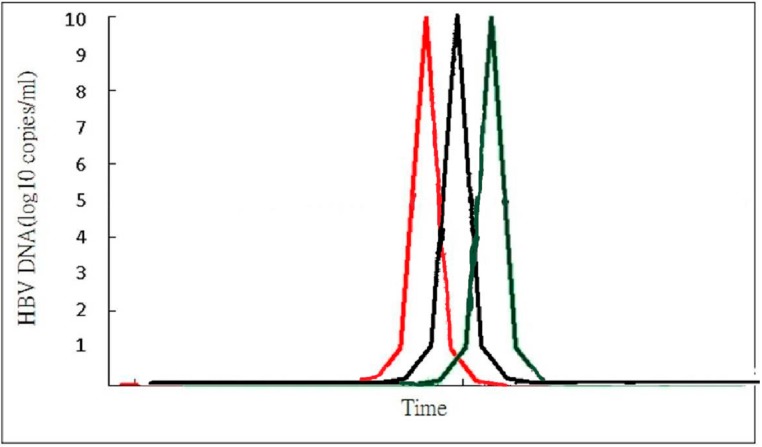
Spontaneous AE of CHB is usually precipitated by reactivated infection, and there is usually an upsurge of serum HBV DNA prior to the abrupt elevation of ALT or bilirubin level. Red line: HBV DNA; Black line: Alanine Aminotransferase (ALT); Green line: Bilirubin.

**Figure 3:**
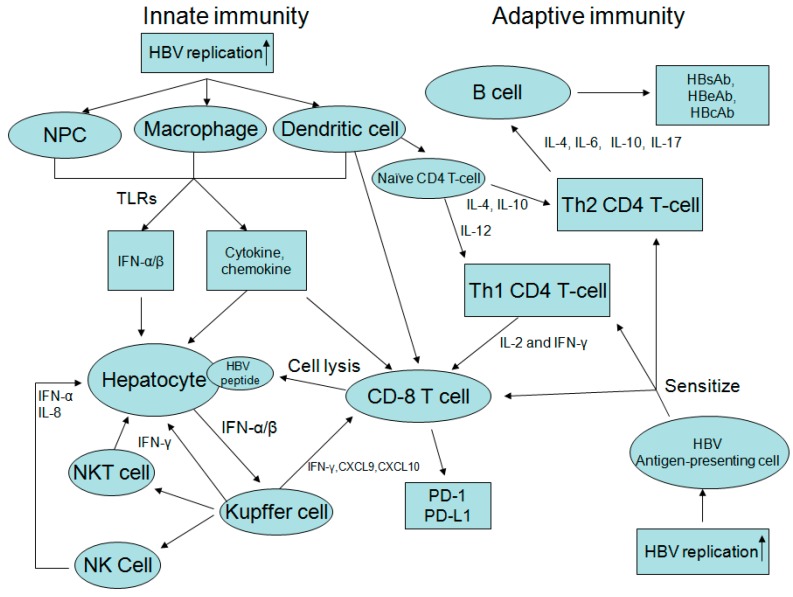
Acute exacerbation of CHB is the result of dynamic changes of both innate and adaptive immune responses. Spontaneous AE of CHB is usually precipitated by reactivated infection, and there is usually an upsurge of serum HBV DNA prior to the abrupt elevation of alanine aminotransferase (ALT) or bilirubin level. Liver injury during these spontaneous AE appears to be mediated by T cells sensitized by HBV antigen presenting cells. Virus-specific CD8+ cytotoxic T cells (with help from CD4+ T cells) can recognize viral antigens presented on infected hepatocytes and lead to direct lysis of the infected hepatocyte. Non-parenchymal cells (NPC), dendritic cells, and macrophages can also produce interferon (IFN) α/β, cytokine and chemokine after recognition of HBV. Increased production of Th1 cytokines, Th2 cytokines, natural killer (NK) cell-mediated pathways, high serum levels of IFN-γ inducible chemokines Chemokine (C-X-C motif) ligand 9 (CXCL)-9 and CXCL-10, programmed cell death protein 1 (PD-1), and its ligand PD-L1, and activation of toll-like receptors (TLR) during AE of CHB are also observed in CHB with AE. IL: interleukin; NKT cell: natural killer T cell; Th1 cell: type I helper T cell; Th2 cell: type II helper T cell.

Acute exacerbation of CHB is the result of dynamic changes of both innate and adaptive immune responses with human leukocyte antigen class I (HLA-I)-restricted, cytotoxic T lymphocyte (CTL)-mediated immune cytolysis of HBV antigen(s) expressing hepatocytes [8,10,37,38]. Spontaneous AE of CHB is usually precipitated by reactivated infection, and there is usually an upsurge of serum HBV DNA prior to the abrupt elevation of alanine aminotransferase (ALT) or bilirubin level [39,40] (Figure 1). The clinical course of CHB with AE can be divided into four stages according to the changes in HBV DNA level (Figure 2). In the ascending limb, HBV DNA <10^5^ copies/mL denotes Stage I while HBV DNA ≥10^5^ copies/mL represents Stage II. In the descending limb, HBV DNA ≥10^5^ copies/mL denotes Stage III while HBV DNA <10^5^ copies/mL represents Stage IV. Patients in Stage I are usually asymptomatic and will seldom seek medical help. Therefore, in clinical practice, patients who visit the hospital due to CHB with spontaneous AE are usually in Stage II, III or IV. If patients visited the doctor at Stage IV, the immune storm due to flare-up of HBV has already been initiated and got exacerbated, which induces the rapid decline of HBV DNA, so the success of antiviral treatment is not anticipated. Moreover, patients in Stage IV have a low HBV DNA level which is also decreasing rapidly, so the benefits of antiviral treatment are expected to be insignificant. Liver injury during these spontaneous AE appears to be mediated by expanded numbers of T cells that are reactive to hepatitis B e antigen (HBeAg) and c antigen (HBcAg) [12,41]. Immunopathologic studies during AE of CHB have shown that the cellular infiltrates at the site of necroinflammatory reaction are mainly CD8 + CTL, which are generally considered to be directed to HBcAg peptides on the surface of hepatocytes [42,43]. Immunologic studies showed a significant elevation of HBcAg/HBeAg-specific precursor T cell, an increase in HBcAg/HBeAg-specific T cell proliferation, a decrease in HBcAg-specific regulatory T cell (Treg) frequencies associated with an increase in HBcAg-specific cytotoxic T lymphocyte (CTL) frequencies. Non-parenchymal cell, dendritic cells and macrophages can also produce interferon α/β, cytokine and chemokine after recognition of HBV. Increased production of Th1 cytokines (interleukin (IL)-2 and IFN-γ), Th2 cytokines (IL-4, IL-6, and IL-10), an increase in IL-17-producing CD4+ T cells, natural killer (NK) cell-mediated pathways (IFN-α and IL-8), high serum levels of IFN-γ inducible chemokines Chemokine (C-X-C motif) ligand 9 (CXCL)-9 and CXCL-10, programmed cell death protein 1 (PD-1) and its ligand PD-L1 during AE of CHB [44,45,46,47,48,49,50]. Cytokine production is associated with activation of toll-like receptors (TLR) and increased expression of TLR-2, TLR-4, TLR-3, TLR-5,TLR-7, TLR-9, TLR-10 are also observed in CHB with AE (Figure 3) (Table 3) [51,52]. However, the event that triggers spontaneous AE of CHB in immune clearance or inactive phase remains unclear.

**Table 3:** Immune profile during spontaneous acute exacerbation of chronic hepatitis B virus (HBV).

Immune Profile	Activity
HBV-specific T cell response	
HBV-specific regulatory T	Decrease
HBV-specific cytotoxic T cell	Increase
NK cell pathway	
IFN-α	Increase
IL-8	Increase
Th1 cytokines	
IL-2	Increase
IFN-γ	Increase
Th2 cytokines	
IL-4	Increase
IL-6	Increase
IL-10	Increase
Chemokines	
CXCL-9	Increase
CXCL-10	Increase
PD-1	Increase
PD-L1	Increase
Toll-like receptors	
TLR-2	Increase
TLR-3	Increase
TLR-4	Increase
TLR-5	Increase
TLR-7	Increase
TLR-9	Increase
TLR-10	Increase

IFN: interferon; IL: interleukin; PD-1: programmed cell death protein 1; PD-L1: programmed death-ligand 1; Tc: cytotoxic T cell; Treg: regulatory T cell; TLR: toll like receptor; CXCL: Chemokine (C-X-C motif) ligand.

## 5. Treatment

Aggressive supportive treatments applied for acute-on-chronic liver failure (ACLF) due to CHB with severe AE include close monitoring of vital signs, fluid status, nutritional status, electrolytes, liver function, antibiotics use for infection, treatment for hepatic encephalopathy, and terlipressin and albumin for hepatorenal syndrome. Extracorporeal liver support systems that replace the detoxification, synthetic, and regulatory functions of the native liver represent a potential solution, but all the devices currently available are still far from ideal [53]. In general, artificial (cell-free) and bioartificial liver support devices have shown their ability to decrease some circulating toxins and to ameliorate hepatic encephalopathy and other intermediate variables. Although they are relatively safe, their effects on the survival of patients with ACLF have not been confirmed [53,54,55]. Recent randomized controlled studies in ACLF patients failed to identify any survival benefit of extracorporeal liver support systems, such as fractionated plasma separation and adsorption (FPSA) and molecular adsorbent recirculating system (MARS) [56,57].

### 5.1. Antiviral Treatment

Interferon therapy is contraindicated in CHB with severe AE as it will cause liver function impairment and exacerbate hepatic decompensation. Nucleos(t)ide analogues (NA) have the profound effect of viral suppression and show good safety profiles in patients with hepatic decompensation, so NA are the drug of choice in CHB with severe AE.

### 5.2. Lamivudine

There has been no randomized study that compared the efficacy of lamivudine *vs.* symptomatic treatment in CHB with severe AE (Table 4). Chan *et al.* [58] in a retrospective study that compared the treatment effect of lamivudine in 28 CHB patients with severe AE *vs.* 18 controls found that six (21.4%) lamivudine-treated patients *vs.* five (27.8%) controls died or received a liver transplant (*p* = 0.62). Multivariate analysis found that platelet ≤1.43 × 10^11^/L and bilirubin >172 micromol/L, but not lamivudine treatment, were independent predictors of liver-related mortality. Similarly, Tsubota *et al.* [59] compared retrospectively the treatment effect of lamivudine in 25 CHB patients with severe AE *vs.* 25 controls. They found hepatic failure developed in six lamivudine-treated patients (24%) and seven controls (28%); and in patients with hepatic failure, three lamivudine-treated patients (12%) and two controls (16%) survived (*p* > 0.15). Lamivudine monotherapy did not prevent progression to hepatic failure or mortality. Multivariate analysis discovered baseline serum bilirubin ≥6 mg/dL, pre-existing cirrhosis, and baseline prothrombin time <40% as independent determinants of rapid progression to hepatic failure. In another retrospective study, Chien *et al.* [16] compared the treatment effect of lamivudine in 60 CHB patients with severe AE *vs.* 31 controls and found that 38% of treated patients and 29% of the controls died (*p* = 0.166). Stepwise logistic regression analysis revealed that both prolonged PT and baseline Child-Pugh scores were significant predictors of mortality, but treatment with lamivudine is not an independent predictor of survival. However, the present study found that of the patients with serum bilirubin <20 mg/dL, all 25 lamivudine-treated patients survived, but five (25%) of 20 untreated patients died (*p* = 0.013). On the contrary, in patients with serum bilirubin level ≥20 mg/dL, the mortality between lamivudine-treated and untreated patients were similar. These results suggest that lamivudine may prevent fatality in CHB patients with hepatic decompensation if therapy starts early enough or before serum bilirubin level rises above 20 mg/dL, which is usually in Stage II or III during CHB with severe AE (Figure 2), but lamivudine helps little if serum levels already exceed 20 mg/dL, which is usually in Stage IV (Figure 2). Sun *et al.* [17] in a matched retrospective cohort study that compared the treatment effect of lamivudine in 130 CHB patients with severe AE *vs.* 130 controls found that the mortality (50.7%, 38/75) of lamivudine-treated patients with MELD scores of 20–30 was lower than that (75.7%, 56/74) of the control group (*p* = 0.002). Moreover, the mortality of lamivudine-treated patients with MELD scores above 30 was 98.0% (48/49) and 100.0% (53/53) in the control group, showing no significant difference between the two groups (*p* = 0.296). A recent meta-analysis showed no benefit of lamivudine *vs.* untreated controls for transplant-free survival in patients with spontaneous severe AE of CHB (OR = 0.98 (95% CI, 0.50–1.92; *p* = 0.956)) [60]. According to the reports of previous studies, lamivudine treatment did not seem to improve survival in CHB with severe AE, but if lamivudine is started early enough before bilirubin level exceeds 20 mg/dL or in patients with less severe liver disease indicated by a MELD score of 20–30, lamivudine treatment is associated with improved survival.

**Table 4:** Lamivudine treatment for chronic hepatitis B with severe acute exacerbation.

Authors	Design	Treatment (Patient Number)	HBV DNA	Mortality (%)	*p*-Value	Prognostic Factors
Chan *et al.* [58]	Retrospective study	LMV (28)	N/A	21.4%	0.62	Platelet
		Control group (18)		27.8%		Bilirubin
Tsubota *et al.* [59]	Retrospective study	LMV (25)	220 *	12%	0.15	Bilirubin
						Cirrhosis
		Control group (25)	120 *	16%		Prothrombin time
Chien *et al.* [16]	Retrospective study	All patients				Prothrombin time
		LMV (60)	22 **	38%	0.166	Child–Pugh scores
		Control group (31)	58.6 **	29%		
		Bilirubin > 20 mg/dL				
		Bilirubin LMV (35)	N/A	66%	NS	
		Control group (11)	N/A	36%		
		Bilirubin < 20 mg/dL				
		LMV (25)	N/A	0%	0.013	
		Control group (20)	N/A	25%		
Sun *et al.* [17]	Retrospective study	MELD: 20–30				LMV treatment
		LMV (76)	86 ***	50.7%	0.002	HBV DNA
		Control group (76)	89 ***	75.7%		Decline of HBV DNA
		MELD > 30				
		LMV (54)	65 ***	98%	0.296	
		Control group (54)	67 ***	100%		

LMV: lamivudine; MELD: the model for end-stage liver disease; N/A: not analyzed; NS: non-significant; * MEq/mL; ** pg/mL; *** Percentage >10^5^ copies/ML.

### 5.3. Entecavir

Several retrospective studies compared the effect of entecavir and symptomatic treatment in CHB with severe AE (Table 5). Chen *et al.* [61] in a retrospective cohort study that compared the treatment effect of entecavir in 55 CHB patients with severe AE *vs.* 74 controls found that 36 (65.5%) entecavir-treated patients *vs.* 55 (74.3%) controls survived for more than three months (*p* = 0.28), although the entecavir-treated group had a significantly greater HBV DNA suppression at 3 months compared with the control group. In a retrospective study that compared the treatment effect of entecavir in 42 CHB patients with severe AE *vs.* 34 controls, Chen *et al.* [62] found that nine (21.4%) in the entecavir-treated group and 20 (58.8%) in the control group died (*p* = 0.007). Ma *et al.* [63] in a retrospective cohort study that compared the treatment effect of entecavir on CHB patients with severe AE *vs.* controls found that 1- and 3-month survival rates of patients in the entecavir-treated group (*n* = 124) were 72.58% and 61.29%, respectively, which were significantly higher than 53.23% and 45.97%, respectively in the control group (*n* = 124) (*p* = 0.022). Survival benefit of entecavir in CHB patients with severe AE has not been proved in randomized controlled studies, although several retrospective studies found that entecavir may achieve better survival than symptomatic treatment. A recent meta-analysis by Zhang *et al.* [64] found that entecavir significantly improved survival at 12 weeks (*p* = 0.0008). Another meta-analysis by Yu *et al.* [65] also found that CHB related ACLD receiving NA including entecavir had significantly lower 3-month mortality (*p* < 0.01) as well as incidence of reactivation (*p* < 0.01). Lange *et al.* [66] found the development of lactic acidosis may likely be the consequence of mitochondrial toxicity in 5 out of 16 patients with cirrhosis and advanced liver disease and a MELD score >20 treated with ETV. So the authors advised caution in administration of ETV in patients with severe liver function impairments. However, the actual risk of lactic acidosis in patients with acute exacerbation or decompensated CHB who received ETV treatment remains controversial and most probably low [67,68].

**Table 5:** Entecavir treatment for chronic hepatitis B with severe acute exacerbation.

Authors	Design	Treatment (Patient Number)	HBV DNA	Mortality (%)	*p*-Value	Prognostic Factors
Chen *et al.* [61]	Retrospective study	ETV (55)	5.7 *	29.5%	0.28	Albumin
						Bilirubin
		Control group (74)	5.1 *	34.5%		Prothrombin time (INR)
						MELD score
Chen *et al.* [62]	Retrospective study	ETV (42)	7.0 **	21.4%	0.007	Bilirubin
						Cholesterol
		Control group (34)	5.7 **	58.8%		Prothrombin activity MELD-Na score
Ma *et al.* [63]	Retrospective study	ETV (124)	6.2	39%	0.022	Bilirubin
						Prothrombin time (INR)
		Control group (124)	6.4	54%		More than 2 comlications
Zhang *et al.* [64]	Meta-analysis	ETV (115)	N/A	43%	0.0008	N/A
		Control group (109)		66%		
Yu *et al.* [65]	Meta-analysis	ETV/LMV (495)	N/A	45%	<0.01	N/A
		Control group (270)		73%		

LMV: lamivudine; MELD: the model for end-stage liver disease; * log copies/mL; ** Log IU/mL; N/A: not analyzed.

### 5.5. Tenofovir

A recent study from India by Garg *et al.* [69] found that the probability of survival in patients with severe spontaneous reactivation of CHB presenting as acute-on-chronic liver failure was higher in the tenofovir than the placebo group (8/14 [57%] *vs.* 2/13 [15%], respectively; *p* = 0.03). Moreover, >2 log reduction in HBV DNA levels at two weeks was found to be an independent predictor of survival (Table 6). The present findings also confirm that if an antiviral agent has profound viral suppression at two weeks, a survival benefit is anticipated. In this study, only patients with HBV DNA levels exceeding 10^5^ copies/mL, who were in Stage II or III but not Stage IV of AE (Figure 2), were enrolled. In patients in Stage IV of AE, the immune storm due to flare-up of HBV has already been initiated and got exacerbated, which induces the rapid decline of HBV DNA, so the success of antiviral treatment is not anticipated (Figure 2). Many previous studies failed to show the benefit of nucleos(t)ide analogue in the treatment of CHB with severe AE, probably because most of these studies enrolled patients not only in Stages II, III and but also Stage IV of AE (Figure 1B).

**Table 6:** Tenofovir treatment for chronic hepatitis B with severe acute exacerbation.

Authors	Design	Treatment (Patient Number)	HBV DNA (IU/mL)	Mortality (%)	*p*-Value	Prognostic Factors
Garg *et al.* [69]	Randomized study	TDF (14)	7.5 × 10^5^	43%	0.03	>2 log reduction in HBV DNA at 2 weeks
		Cotnrol group (13)	1.7 × 10^6^	85%		

TDF: tenofovir.

### 5.6. Treatment Efficacy of Different Nucleos(t)ide Analogues

#### 5.6.1. Lamivudine *vs.* Entecavir

Comparison of the treatment efficacy of lamivudine *vs.* entecavir is shown in Table 7. Cui *et al.* [70] in a retrospective study that compared the treatment effect of entecavir in 33 CHB patients with severe AE *vs.* that of lamivudine in 34 counterparts found that 48.5% entecavir-treated *vs.* 50% lamivudine-treated patients survived for more than three months (*p* = 0.72). Chen *et al.* [61] in a retrospective study that compared the treatment effect of entecavir in 42 CHB patients with severe AE *vs.* that of lamivudine in 30 counterparts found that three-month mortality was 33% in entecavir-treated *vs.* 40% in lamivudine-treated patients (*p* = 0.374). Lai *et al.* [71] in a retrospective study that compared the treatment effect of entecavir in 93 CHB patients with severe AE *vs.* that of lamivudine in 89 counterparts found that the mortality rate was 91.7% in entecavir-treated *vs.* 92% in lamivudine-treated patients (*p* = 0.680). Liu *et al.* [72] in a retrospective study that compared the treatment effect of entecavir in 31 CHB patients with severe AE *vs.* that of lamivudine in 34 counterparts found that the mortality rate was 0% in entecavir-treated *vs.* 3% in lamivudine-treated patients (*p* = 0.385). Zhang *et al.* [73] in a retrospective study that compared the treatment effect of entecavir in 65 CHB patients with severe AE *vs.* that of lamivudine in 54 counterparts found that 51 (78.5%) in the entecavir group and 35 (64.8%) in the lamivudine group survived at day 60 (*p* = 0.066). Chen *et al.* [74] in a retrospective study that compared the treatment effect of entecavir in 107 CHB patients with severe AE *vs.* that of lamivudine in 215 counterparts found that the overall mortality in the entecavir and lamivudine groups at 24 week was 21.2% and 12.3%, respectively (*p* = 0.02). However, in the present study, the lamivudine group had a significantly lower albumin level and a higher MELD score at baseline. In addition, multivariate analysis did not identify entecavir treatment as an independent factor associated with survival. But the entecavir group achieved better virological response than the lamivudine group at week 24 and 48. Wong *et al.* [75] in a retrospective study that compared the treatment effect of entecavir in 36 CHB patients with severe AE *vs.* that of lamivudine in 117 counterparts found that seven (19%) patients in the entecavir group and five (4%) patients in the lamivudine group died (*p* = 0.010). Multivariate analysis also identified entecavir treatment as an independent factor associated with mortality. But entecavir treatment resulted in more rapid and complete viral suppression, with more patients achieving undetectable HBV DNA at week 48, compared to the lamivudine group. Previous studies by Chien *et al.* [16] and Sun *et al.* [17] have found that if lamivudine was given in an earlier stage of severe AE, such as in Stage II or III (Figure 2), a survival benefit could be attained. Most of the previous studies that compared the efficacy of lamivudine and entecavir enrolled patients with mild and severe liver disease in Stages II, III and also Stage IV of AE (Figure 2). In a recent retrospective study, Tsai *et al.* [76] compared the treatment effect of entecavir *vs.* lamivudine in CHB patients with severe AE having HBV DNA levels above 10^5^ copies/mL and bilirubin levels below 15 mg/dL. They found that 5 out of 40 patients (12.5%) in the entecavir group and 1 out of 59 patients (1.7%) in the lamivudine group died. Multivariate analysis found that entecavir treatment was associated with more mortality than lamivudine (*p* = 0.035). Early entecavir treatment for CHB with severe AE seemed to have a higher mortality than lamivudine treatment. Ye *et al.* [77] in a recent meta-analysis of 12 randomized controlled studies that compared treatment of lamivudine (*N* = 450) with entecavir (*N* = 423) in decompensated HBV cirrhosis found that, despite the better suppression of viral load in entecavir recipients, the mortality rate in lamivudine and entecavir recipients with decompensated cirrhosis was similar ranging between 7.89% and 6.37% respectively. In this meta-analysis the safety record for both anti-viral agents was similar. Another meta-analysis by Yu *et al.* [65] also found that there is no difference in short term mortality in patients treated with entecavir or lamivudine (36.4% *vs.* 40.4% respectively) in HBV-related acute-on-chronic liver failure. According to previous findings, there is no firm conclusion on whether entecavir or lamivudine treatment promises a better outcome for CHB with severe AE. Further randomized study that compare the efficacy of lamivudine *vs.* entecavir is required. However, lamivudine is limited by its high rate of resistance in the treatment of CHB [78,79]. Long-term follow-up study also found that lamivudine treatment for CHB with SAE resulted in a high rate of drug resistance and virological breakthrough [80]. Current AASLD (2009) and EASL (2012) guidelines do not recommend a specific NA for treatment of decompensated chronic liver disease or acute exacerbation, although there is a consensus that suggested treatment with a potent anti-viral agent [81,82]. Lamivudine is not inferior to entecavir in the treatment of CHB with severe AE, but lamivudine is cheaper than entecavir and is still widely used in many countries, especially in those with poor economic condition. Early short-term lamivudine use to prevent resistance followed by potent NA such as tenofovir may be another treatment option.

**Table 7:** Comparison of the treatment outcome of lamivudine and entecavir in chronic hepatitis B with severe acute exacerbation.

Authors	Design	Treatment (Patient Number)	HBV DNA (Log copies/mL)	Mortality (%)	*p*-Value	Prognostic Factors
Cui *et al.* [70]	Retrospective study	ETV (33)	5.9	51.5%	0.72	Age
		LMV (34)	5.9	50%		cholinesterase
						MELD score
Chen *et al.* [61]	Retrospective study	ETV (42)	6.4	51.5%	0.374	Bilirubin
		LMV (30)	5.6	50%		Cholesterol
						Prothrombin activity MELD-Na score
Lai *et al.* [71].	Retrospective study	ETV (93)	6.4	51.5%	0.680	Bilirubin
		LMV (89)	5.6	50%		Creatinine
						Prothrombin time MELD score
Liu *et al.* [72]	Retrospective study	ETV (31)	6.2	0%	0.385	N/A
		LMV (34)	7.0	3%		
Zhang *et al.* [73]	Retrospective study	ETV (65)	7.0	21.5%	0.066	Gender
						HBeAg(+)
						MELD score
						Child–Pugh scores
		LMV (54)	7.2	35.2%		Undetectable HBV at 30 days
Chen *et al.* [74]	Retrospective study	ETV (107)	6.5	21.2%	0.02	MELD score
		LMV (215)	6.5	12.3%		Ascites
						Hepatic enceophalopathy
Wong *et al.* [75]	Retrospective study	ETV (36)	7.3	19%	0.010	Prothrombin time
		LMV (117)	7.6	4%		ETV treatment
Tsai *et al.* [76]	Retrospective study	ETV (40)	8.3	12.5%	0.035	Prothrombin time
		LMV (59)	8.4	1.7%		ETV treatment
Ye *et al.* [77]	Meta-analysis	ETV (423)	N/A	6.4%	NS	N/A
		LMV(450)		7.9%		
Yu *et al.* [65]	Meta-analysis	ETV (192)	N/A	36.4%	0.35	N/A
		LMV (148)		40.5%		

ETV: entecavir; LMV: lamivudine; MELD: the model for end-stage liver disease; HBeAg: hepatitis B e antigen; N/A: not analyzed; NS: non-significant.

#### 5.6.2. Entecavir *vs.* Tenofovir

There is only one study that compared the efficacy of entecavir *vs.* tenofovir in CHB with severe AE (Table 8). Hung *et al.* [83] in a retrospective study that compared the treatment effect of entecavir in 148 CHB patients with severe AE *vs.* that of tenofovir in 41 counterparts found that 23 (16%) patients in the entecavir group and 7 (17%) patients in the tenofovir group died or received liver transplantation (*p* = 0.749). Tenfovir and entecavir produce similar treatment responses and clinical outcomes in CHB patients with severe AE. Although tenofovir and entecavir seemed to have similar efficacy in the treatment of CHB with severe AE, a further randomized study that compared the efficacy of tenofovir *vs.* entecavir is required.

**Table 8:** Comparison of the treatment outcome of tenofovir and entecavir in chronic hepatitis B with severe acute exacerbation.

Authors	Design	Treatment (Patient Number)	HBV DNA (Log copies/mL)	Mortality (%)	*p*-Value	Prognostic Factors
Hung *et al.* [83]	Retrospective study	ETV (148)	6.5	16%	0.797	Hypertension
						HBV DNA
						Platelet
						MELD score
						Ascites
						Hepatic encephalopathy
		TDF (41)	7.0	17%		Hepatorenal syndrome

ETV: entecavir, TDF: tenofovir, MELD: the model for end-stage liver disease.

#### 5.6.3. Treatment Emergence Mutants

Nucleos(t)ide analogue treatment for chronic HBV is usually associated with the development of resistance mutant. Lamivudine treatment is associated with a resistance rate of 15%–25% per year and at five years up to 70% of patients may develop lamivudine resistance mutants [84,85]. Resistant viruses show a characteristic mutation of the 550th amino acid methionine in the tyrosine-methionine-aspartate-aspartate (YMDD) motif of DNA polymerase to isoleucine (YIDD) or valine (YVDD) [86,87]. Among chronic HBV patients with severe acute exacerbation treated with lamivudine, virological breakthrough is common [80]. Wong *et al.* [80] in a long-term follow-up study found that lamivudine treatment in patients with severe acute exacerbation had higher HBeAg seroconversion rates and lower risks of virological breakthrough. However 33% of patients developed lamivudine resistance mutant in five years. Among the fifteen patients with lamivudine resistance mutants, six had rtM204I, one had rtM204V, one had both rtM204I and rtM204V mutations and seven patients had rtL180M mutation. Another study by Akuta *et al.* [88] found the cumulative occurrence rates of YMDD mutations in SAE and non-SAE groups were 5.6% and 19.3% at one year, 34.5% and 37.3% at two years, and 34.5% and 37.3% at three years, respectively and the emergence of YMDD mutations tended to happen later in the SAE group than in the non-SAE group. Zang *et al.* [89] in chronic HBV with acute exacerbation under lamivudine treatment found that apart from mutations at the YMDD motif, no shared mutations were shown and strains with high replicative activity might be selected from the total HBV quasispecies during treatment, and amongst these strains, those with core promoter mutations were most likely to be related to severe clinical exacerbations. Resistance to entecavir in HBV appears to occur through a two-hit mechanism with initial selection of M204V/I mutation followed by amino acid substitutions at rtT184, rtS202, or rtM250 [90]. However resistance related to entecavir treatment in CHB is extremely low [81,82]. To date, primary resistance to tenofovir in patients with CHB mono-infection has also never been reported [81,82]. A study of CHB and HIV co-infected patients suggested a possible role of rtA194T mutation in tenofovir resistance [91]. HBV with the rtA194T mutation was shown to have a reduced susceptibility to tenofovir when combined with lamivudine resistance rtM204V and rtL180M mutations *in vitro* [92]. The clinical impact of the rtA194T mutation remains to be determined; tenofovir has been found to be effective in restricting the replication of HBV in patients with the rtA194T mutation [92]. The hepatitis B virus (HBV) polymerase and envelope genes overlap in such a way that resistance mutations to antiviral agents in the reverse transcriptase gene may influence the antigenicity of the HBV surface antigen [93]. Two types of surface proteins mutants are recognized. The first type occurred due to amino acids substitutions caused by primary and compensatory resistance mutations in the polymerase gene, which generates S gene mutations and the second type arose due to prolonged viral suppression leading to seroclearance of surface antigen, where vaccine-escape-like mutations might be selected [93]. A triple mutations (rtV173L + rtL180M + rtM204V) causing lamivudine resistance has recently been shown to enhance HBV replication, compared with rtL180Mt + rtM204V alone [94]. This triple HBV mutant resulted in two amino acid changes in the overlapping surface gene (sE164D + sI195M), which decrease anti-HBs binding to levels seen only with the vaccine escape mutant sG145R [95,96]. Selection of an sP120A mutation in CHB patients treated with lamivudine is also associated with the apparent HBsAg seroconversion and this mutation produces a reduced anti-HBs binding, which explains the failure to detect HBsAg [97]. Yeh *et al.* [98] discovered in patients treated with lamivudine who developed an rtA181T mutation that in an *in vitro* phenotypic assay was confirmed to be associated with lamivudine resistance and this mutation generates a stop codon in the surface antigen (sW172stop), which causes decreased secretion of the HBsAg and decreased viral fitness. Interestingly, neither the adefovir-associated resistance mutation rtN236T nor the tenofovir-associated resistance mutation rtA194T causes changes in the HBV surface gene [99]. Sloan *et al.* [100] in CHB treated with lamivudine, adenofovr and entecavir found that the mutations rtF166L/sF158Y (lamivudine-related, compensatory) and rtl169T/sF161L (entecavir-related, primary) acting alone, and the mutations rtV173L/sE164D (lamivudine-related, compensatory) and rtSilent/sD144E (antibody escape-related) each when combined with rtM204V/sl195M (lamivudine-associated, primary) resulted in decreases in antibody reactivity to epitopes in the first or second loop, or in both loops. HBV was also found to be able to develop the commensurate rtV173L mutation in the polymerase protein, which restore the replication phenotype of lamivudine resistant HBV [94,101].

## 6. Prognostic Factors

The mortality rate of CHB with severe AE after introduction of antiviral agents has been examined. In patients who received lamivudine, the mortality rate ranged from 12%–98% according to the severity of disease at exacerbation (Table 2). Dai *et al.* [102] in a study of 96 patients of CHB with severe AE who received lamivudine treatment found that The MELD and Index scoring systems were good predictors of 6-month survival. In patients who received lamivudine before bilirubin rose above 20 mg/dL, the mortality rate was 0%, whereas if lamivudine was started after bilirubin exceeded 20 mg/dL, the mortality rate increased to 66% [16]. In patients with MELD scores of 20–30, lamivudine treatment had a mortality rate of 50.7% but in patients with MELD scores exceeding 30, the mortality rate rose to 98% [17]. The prognostic factors associated with lamivudine treatment included platelet count, bilirubin level, prothrombin time, cirrhosis of liver and Child-Pugh scores (Table 4). In patients who received entecavir, the mortality rate ranged from 21% to 51.5%. Yan *et al.* [103] in a retrospective study that evaluated the prognostic factors of entecavir treatment of 109 CHB patients with severe AE found that MELD score ≥30 predicted very poor prognosis due to fatal liver failure. The prognostic factors associated with entecavir treatment included age, albumin, bilirubin, prothrombin time, ascites, hepatic encephalopathy and MELD score (Table 5). In patients who received tenofovir, the mortality rate ranged from 17% to 43%. The prognostic factors associated with tenofovir treatment included >2 log reduction in HBV DNA at two weeks, hypertension, HBV DNA, platelet count, MELD score, ascites, hepatic encephalopathy, and hepatorenal syndrome (Table 6 and Table 7). In general, liver reserve on presentation including albumin, bilirubin, and prothrombin time and MELD score is the major prognostic factor in CHB with severe AE under antiviral treatment.

An early and accurate prognostic system based on the integration of laboratory indicators, clinical events and some mathematic logistic equations is needed to optimize treatment for patients of CHB with SAE. Several scoring systems have been developed to predict the prognosis of CHB with severe AE. The MELD score was the most common and the donor-MELD was the most innovative for patients on the waiting list for liver transplantation [104,105]. The guideline of the Asian Pacific Association for the Study of the Liver (APASL) recommended that patients with HBV reactivation with intermediate MELD should be assessed for early transplant if cirrhosis, bilirubin > 10 mg/dL, PT < 40%, and platelet < 1.00 × 10^11^/L [106]. Greater than 2 log reduction in HBV DNA at 2 weeks is the most important on-treatment prognostic factor in CHB with SAE. The role of HBV DNA as an independent prognostic factor in CHB with severe AE is controversial. Many studies did not confirm the prognostic role of HBV DNA [16,58,59,61,62,63]. Only few studies identified HBV DNA as an independent prognostic factor in CHB with severe AE undergoing antiviral treatment. Hung *et al.* [83] in a retrospective study that compared the treatment effect of entecavir and tenofovir in CHB with severe AE found that baseline HBV DNA is an independent factors for mortality or liver transplantation. Hsu *et al.* [107] in another retrospective study of 66 CHB patients with severe AE who received antiviral treatment found that pretreatment HBV DNA level stratified the risk of death. In a study on lamivudine treatment for CHB with severe AE, Chien *et al.* [16] found that patients with undetectable serum HBV DNA levels had significantly higher bilirubin levels than those with detectable serum HBV DNA. Apart from this, similar clinical features and mortality rates were observed between those with undetectable and detectable serum HBV DNA levels in both groups (5/11 or 45% *vs.* 18/49 or 37% in the lamivudine treated group and 0/3 or 0% *vs.* 10/28 or 36% in the control group; *p* > 0.05). The role of HBV DNA as an independent prognostic factor in CHB with severe AE undergoing antiviral treatment has not been confirmed.

## 7. Conclusions

CHB with spontaneous AE is not uncommon. These exacerbations can be mild, but some patients may develop hepatic decompensation and even die. The underlying pathogenesis of CHB with spontaneous AE is a dynamic process of immune response between HBV and the host. The benefit of nucleos(t)ide analogues (NA) treatment in CHB with severe AE is controversial. Early lamivudine treatment before the bilirubin level exceeded 20 mg/dL or a MELD score below 30 seemed to be associated with an improved outcome. Tenofovir has also been found to be associated with better outcomes in CHB with severe AE with a high HBV DNA level. The survival benefit of different NAs has not been confirmed in a randomized study in the setting of CHB with severe AE. However, early NA use with potent agents and good resistance profile is recommended in all CHB patients with severe AE.
